# Clinical and Diagnostic Findings in Dogs Infected with *Trichuris vulpis*: A Retrospective Study

**DOI:** 10.3390/vetsci11070306

**Published:** 2024-07-10

**Authors:** Mario Cervone, Marine Hugonnard, Gilles Bourdoiseau, Luc Chabanne, Emilie Krafft, Jean-Luc Cadoré

**Affiliations:** 1 Département des Animaux de Compagnie, de Loisir et de Sport, Campus Vétérinaire de Lyon, Université de Lyon, VetAgro Sup, 1 Av. Bourgelat, 69280 Marcy L’Etoile, France; marine.hugonnard@vetagro-sup.fr (M.H.);; 2 Département Elevage et Santé Publique, Université de Lyon, VetAgro Sup, Campus Vétérinaire de Lyon, 69280 Marcy L’Etoile, France

**Keywords:** anemia, hypoalbuminemia, pseudo-hypoadrenocorticism, whipworms

## Abstract

**Simple Summary:**

This retrospective study aimed to compare clinical and diagnostic features of *Trichuris vulpis*-infected dogs with those also harboring other intestinal parasites. Forty-five dogs were included, twenty-five solely infected with *T. vulpis* (categorized as G1) and twenty with concurrent infections (categorized as G2). Weight loss was more frequent in G2 than in G1 (*p* = 0.006). No significant differences were observed in laboratory abnormalities between G1 and G2. Only diarrhea was more frequent in younger dogs compared to adults (*p* = 0.007), while adult dogs had higher egg shedding compared to young ones (*p* = 0.04). A significant positive correlation (r = 0.41; *p* = 0.005) between egg shedding and age was also found. These findings suggest *T. vulpis* may cause clinical signs in dogs regardless of age or co-infection.

**Abstract:**

*Trichuris vulpis* is a parasite of the large intestine of canids and has a global distribution. Despite its well-established epidemiology, the question of its pathogenicity in dogs remains debated. It has been suggested that younger age and concurrent infection with *Ancylostoma caninum* may be responsible for more severe clinical presentations. This retrospective study aimed to describe the clinical and diagnostic features of *T. vulpis*-infected dogs and to compare these findings with dogs infected with both *T. vulpis* and other intestinal parasites (poly-infected dogs). Forty-five dogs were included, with twenty-five being solely infected by *T. vulpis* and twenty poly-infected dogs. Only weight loss was more frequent (*p* = 0.006) in poly-infected dogs compared to *T. vulpis* mono-infected dogs. No significant differences were observed in laboratory abnormalities between mono-infected and poly-infected dogs. Only diarrhea was more frequent (*p* = 0.007) in younger dogs compared to adults. The egg shedding pattern was significantly higher (*p* = 0.04) among adult dogs compared to young ones, and there was a significant positive correlation between egg shedding and age (r = 0.41; *p* = 0.005). These findings suggest that *T. vulpis* might be responsible for both clinical signs and laboratory abnormalities in dogs, irrespective of the host’s age and the presence of other intestinal parasites.

## 1. Introduction

Intestinal nematodes belonging to the genus *Trichuris* typically inhabit the caecum and colon of various mammals, including humans [1,2,3]. They are commonly known as whipworms due to their distinctively thick posterior body end. Recent morphological investigations have also described a specific region at their anterior body end, termed the bacillary band, which consists of cuticular inflations, stichocytes, and bacillary glands serving both secretory and absorptive functions [4,5].

*Trichuris vulpis*, a species within this genus, infects both domestic and wild canids, and is one of the most frequently reported gastrointestinal (GI) nematodes in dogs, especially in kenneled and shelter dogs [1,6]. *Trichuris vulpis* has a worldwide distribution, with reported prevalence in Europe ranging from 0.8% up to 17.6% [7,8,9]. In France, an apparent prevalence of 2.7% has been recently reported [10]. Infection occurs through the ingestion of embryonated eggs that are difficult to eliminate from contaminated soil and water, thus exposing dogs to continuous re-infection [6]. After ingestion, infective larvae emerge from embryonated eggs and penetrate the intestinal glands for up to two weeks for molting [1,6]. Thereafter, *T. vulpis* colonizes the large intestine and its location varies from the cecum to the colonic mucosa depending on the worm burden [6]. The prepatent period ranges from 8 to 12 weeks [1].

*Trichuris vulpis* has also been sporadically isolated from human feces, sometimes associated with GI signs. Additionally, cases of presumed human visceral *larva migrans* caused by *T. vulpis* have been reported in both pediatric and adult patients. However, the zoonotic potential of *T. vulpis* still remains debated [1]. 

The pathogenicity of *T. vulpis* in dogs remains a subject of controversy [1]. Experimental studies in dogs have demonstrated a wide range of outcomes, from minimal tissue damage indicating a high host tolerance to infection, to localized inflammation, hemorrhaging, and even fatal outcomes [1,11,12,13,14]. While adult dogs often carry a higher parasite burden, subclinical disease is more frequent in this group. Conversely, clinical manifestations, including diarrhea, hyporexia, dehydration, lethargy, and weight loss, are more commonly observed in young dogs [1,3,15]. Some studies have also reported severe clinical syndromes resembling hypoadrenocorticism, characterized by decreased sodium/potassium (Na/K) ratios despite normal ACTH-stimulation test results [16,17,18,19]. It has been suggested that clinical and laboratory abnormalities associated with *T. vulpis* infection are influenced by several factors, including the age of the host and the parasitic burden, as well as concurrent infections with other parasitic species, notably *Ancylostoma caninum* co-infection [1,13,14,20].

This retrospective study aimed to describe the clinical and laboratory findings in dogs infected with *T. vulpis* and to compare these features between dogs solely infected with *T. vulpis* and those concurrently infected with *T. vulpis* and other parasitic species.

## 2. Materials and Methods

The canine computing medical database of the Veterinary Teaching Hospital of VetAgro Sup (Lyon, France) was retrospectively reviewed for dogs diagnosed with *T. vulpis* infection between January 2002 and December 2018. Dogs were included if fecal flotation with a zinc sulfate solution (specific gravity 1.20) revealed the presence of *T. vulpis* eggs and were therefore categorized as group 1 (G1; dogs infected with *T. vulpis* alone) and group 2 (G2; poly-infected dogs). Poly-infected dogs were designated as dogs harboring *T. vulpis* and other different endoparasites, including both GI (helminths and protozoa) and non-GI endoparasites. The diagnosis of a *T. vulpis* infection was based on size, plug aspects, and shell wall surface pattern of eggs with a “lemon-shape”, while using previously described diagnostic keys for distinguishing *T. vulpis* eggs from those of *Eucoleus aerophilus* (syn. *Capillaria aerophila*) and *Eucoleus boehmi* [21]. Briefly, *T. vulpis* eggs were morphologically recognized by their larger size (70–80 μm by 30–42 μm), their symmetrical shape, the presence of salient polar plugs aligned on opposite sides, and the smooth shell surface [21]. The adult burden of *T. vulpis* was semi-quantitatively estimated by the number of *T. vulpis* eggs per 5 g of feces. Fecal shedding patterns (FE) of *T. vulpis* eggs were categorized as “weak” (≤10 eggs/5 g of faces); “mild” (11–100 eggs/5 g feces); “moderate” (101–1000 eggs/5 g of faces); and “massive” (>1000 eggs/5 g of feces), although this estimation was approximate as quantitative techniques like the McMaster method were not used. Furthermore, these intervals and classifications are subjective, and solely used in our hospital to have an approximative semi-quantitative estimation.

Clinical signs and laboratory results were recorded. Cases diagnosed with concurrent diseases (excluding coexisting intestinal parasitic infections) that could potentially contribute to GI signs or laboratory abnormalities were excluded. For each dog, signalment and clinical signs including diarrhea, hematochezia, weight loss, vomiting, hyporexia, and polyphagia were recorded. Digestive signs were classified as acute (lasting less than 10 days) and chronic (lasting more than 10 days). Previous administration of antiparasitic treatments was noted, with dogs considered “dewormed” if they had received antiparasitic drugs within the preceding six months or if such drugs were routinely administered at least twice a year. The results of complete blood count (CBC), biochemical analyses (including serum albumin, sodium, chloride, and potassium concentrations, as well as ACTH-stimulation test results), and diagnostic imaging findings were recorded when available. Anemia was defined as regenerative when reticulocyte count was >60,000 mcL. Corrected hypochloremia was calculated as previously described when hyponatremia was present [17].

### Statistical Methods

Statistical analysis was conducted using commercially and online available software (XLSTAT, Data Analysis and Statistical Solution for Microsoft Excel (version 16.86); Social Science Statistics www.socscistatistics.com, accessed on 26 June 2023). Hypothesis tests were two-tailed, and the level of significance was set at *p* < 0.05.

Data were assessed for normality using the Kolmogorov–Smirnov test. For descriptive statistics, data are presented as mean ± standard deviation (SD) and median with range. Numerical data were compared between G1 and G2 using the Student’s *t*-test for normally distributed data and the Kruskal–Wallis test for non-normally distributed data. A chi-square analysis or Fisher’s exact test was performed to compare signalment characteristics, previous deworming administration, FE patterns, frequency of GI signs, laboratory test abnormalities, and the duration of GI signs (acute versus chronic) between groups (G1 versus G2), as well as between dogs categorized as young (≤one year of age) and adult (>one year of age), when applicable. The frequency of GI signs and laboratory test abnormalities were also compared between dogs with different FE patterns. For categorical data comparison, the FE pattern was broadly categorized as “low” (≤100 eggs/5 g of feces) and “high” (≥101 eggs/5 g of feces). To evaluate the correlation between the FE pattern and age, CBC, and biochemistry parameters, a linear regression analysis with a calculation of Spearman’s coefficient (r) was performed.

## 3. Results

Between January 2002 and December 2018, a total of 49 dogs had eggs of *T. vulpis* in their feces at fecal flotation. Three of these dogs were excluded from the study due to concomitant diagnoses of protein-losing enteropathy (one), alimentary lymphoma (one), and disseminated histiocytic sarcoma (one). Additionally, one dog was excluded due to an uncertain final diagnosis. Consequently, 45 dogs were ultimately included in the study. Among them, 25 were solely infected by *T. vulpis* (G1), while 20 were poly-infected dogs (G2). Other GI parasites included *Toxocara canis* (55%), Ancylostomatidae (40%), *E. aerophilus* (10%), Tæniidae (5%), *Cystoisospora* spp. (35%) and *Giardia duodenalis* (20%) (Table 1). Within the group of poly-infected dogs, eleven were concurrently infected by two parasites, six by three parasites, two by four parasites, and only one dog by five parasites.

### 3.1. Signalment

Out of the 45 dogs enrolled in the study, 3 were of mixed breed, while 23 were purebred. Purebred dogs included Beagle (five), labrador retriever (four), golden retriever (three), cocker spaniel (three), German shepherd (three), Siberian Husky (two), Dachshund (two), Beauceron (two), Finnish Lapphund (two), Drahthaar (two), Brittany spaniel (two), Langhaar (one), border collie (one), springer spaniel (one), English setter (one), Weimaraner (one), Khortal (one), Bleu de Gascogne (one), Bruno Jura hound (one), Pointer (one), Rottweiler (one), Yorkshire terrier (one) and Scottish terrier (one). Eleven dogs resided in multi-dog households (five in G1 and six in G2). The age of the dogs ranged from four months to fourteen years, with a median age of four years and a mean of 4.9 years ± 3.9. Within G1, the median age was 4.5 years (mean of 5.9 years ± 4.4), whereas in G2, it was 3.5 years (mean of 3.6 years ± 2.8). Dogs in G2 were found to be younger compared to those in G1 (*p* = 0.02). Twelve dogs were aged ≤ one year (considered as young dogs), while thirty-three dogs were aged > one year (considered as adult dogs).

### 3.2. Fecal Shedding Pattern

Based on fecal flotation results, the overall pattern of *T. vulpis* FE was categorized as weak in 12 dogs (27%), mild in 14 dogs (31%), moderate in 8 dogs (18%), and massive in 11 dogs (24%). There was no statistically significant difference in the FE pattern between G1 and G2 (*p* = 0.25). The FE pattern of *T. vulpis* eggs was notably higher (*p* = 0.04) among adult dogs in comparison to young dogs, with young dogs consistently displaying no massive FE pattern. Additionally, there was a weak yet statistically significant positive correlation (r = 0.41; *p* = 0.005) between the age of the dogs and the FE pattern.

### 3.3. Antiparasitic Prophylaxis

Information regarding deworming was available for 39 out of 45 dogs. Among these, a total of 32 dogs (71%) were classified as dewormed, with 17 in G1 and 15 in G2, showing no statistical difference between the groups. Conversely, seven dogs (15%) were not dewormed (five in G1 and two in G2), also showing no statistical difference. Information was not available for six dogs (three in each group). Additionally, no statistical difference was observed regarding the FE pattern between dewormed and untreated dogs (*p* = 0.32).

### 3.4. Clinical Signs

Clinical signs were present in 42/45 dogs (93%). Gastrointestinal signs were recorded in 34 dogs (75%), including diarrhea in 22 (49%), weight loss in 17 (38%), hematochezia in 13 (29%), hyporexia in 13 (29%), vomiting in 8 (18%), and polyphagia in 4 (9%) (Table 2). Notably, weight loss appeared to be more frequent (*p* = 0.006) in dogs in G2 (60%) compared to those in G1 (20%). Overall, young dogs presented with diarrhea more frequently than adults (83% and 36%, respectively; *p* = 0.007). This finding was also confirmed when only *T. vulpis* mono-infected dogs were considered (100% of young dogs compared to 25% of adult dogs; *p* = 0.005). Among the dogs presenting GI signs, 20 had chronic signs (62%). The duration of clinical signs was not documented in two dogs. There was no statistical difference observed between G1 and G2 regarding the duration of GI signs (*p* = 0.46). Eight dogs (18%) did not manifest GI signs (six in G1 and two in G2) but exhibited other clinical abnormalities including coughing (four), lethargy (two), nasal discharge (one), and seizures (one). Fecal analysis was performed as part of the diagnostic assessment in these cases. Three dogs (7%) were asymptomatic (one in G1 and two in G2), and fecal analysis was conducted as a screening test. Dogs with different FE patterns had similar clinical signs (*p* = 0.70).

### 3.5. Laboratory Results

#### 3.5.1. Complete Blood Count Results

Complete blood count results were available for 30 dogs (19 dogs in G1 and 11 dogs in G2) (Table 3). However, data on red blood cell (RBC) indices and white blood cell (WBC) differential counts were unavailable for three and four dogs, respectively. Anemia was identified in 23% of cases (five dogs in G1 and two dogs in G2), with a median hemoglobin (Hg) concentration of 10 g/dL, ranging from 6.5 to 11.5 (mean of 9.1 ± 2.1). Notably, the two anemic dogs in G2 were concurrently infected with Ancylostomatidae (one dog) and Ancylostomatidae, *E. aerophilus*, and *Cystoisospora* spp. (one dog). Of the seven anemic dogs, only two (29%, both from G1) had severe anemia (Hg concentration of 6.5 g/dL and 6.7 g/dL, respectively). Interestingly, lethargy was the sole reported clinical abnormality in these two dogs, while two additional dogs (both from G1) also displayed concurrent hematochezia. Overall, anemia was regenerative in four dogs (57%), with a median absolute reticulocyte count of 103 × 10^9^/L, ranging from 74.4 to 105 (mean of 108.8 ± 33.6). Anemia was macrocytic (MCV of 79 fL) in one dog and microcytic (MCV of 45 fL and 51 fL, respectively) in two dogs. In the two dogs with severe regenerative anemia, an exhaustive diagnostic evaluation (including diagnostic imaging, biochemistry, and direct Coombs test) yielded no abnormalities.

Neutrophilic leukocytosis was recorded in 30% of dogs (five dogs in G1 and three dogs in G2), with a median WBC count of 23.8 m/mm^3^, ranging from 19.4 to 57.0 (mean of 31.7 m/mm^3^ ± 16). Eosinophilia was observed in 35% of dogs (six dogs in G1 and three in G2), with a median eosinophil count of 1.12 m/mm^3^, ranging from 0.57 to 1.96 (mean of 1.1 m/mm^3^ ± 0.5). Lymphopenia was identified in 46% of dogs (nine dogs in G1 and three dogs in G2) with a median lymphocyte count of 0.7 m/mm^3^, ranging from 0.3 to 0.89 (mean of 0.6 m/mm^3^ ± 0.2), while monocytosis was observed in 8% of dogs (two dogs in G2), with monocyte counts of 2.15 m/mm^3^ and 5.65 m/mm^3^, respectively. None of the dogs exhibited thrombocytopenia, while mild to moderate thrombocytosis was noted in 11% of dogs (two in G1 and one in G2), with a median platelet count of 894 m/mm^3^, ranging from 631 to 978 (mean of 834 m/mm^3^ ± 181). No statistical difference was found in CBC results and the frequency of CBC abnormalities between dogs in G1 and G2 (Table 3). A weak but still statistically significant positive correlation was observed between the FE pattern and the total WBC count (r = 0.53; *p* = 0.003) and neutrophil count (r = 0.55; *p* = 0.003), as well as a weak but still statistically significant negative correlation between the FE pattern and the lymphocyte count (r = −0.49; *p* = 0.01). However, no correlation was found between the FE pattern and the eosinophil count (r = −0.21; *p* = 0.29) or monocyte count (r = 0.02; *p* = 0.93), hemoglobin concentration (r = −0.11; *p* = 0.95), or platelet count (r = 0.18; *p* = 0.35).

#### 3.5.2. Biochemistry Results

Biochemistry results were available for 21 dogs (14 in G1 and 7 in G2), including serum electrolyte concentrations in 14 dogs (11 in G1 and 3 in G2).

Hypoalbuminemia (median 24.5 g/L, ranging from 18 to 26; mean of 23.6 g/dL ± 2.5) was observed in 12 dogs (57%; 8 in G1 and 4 in G2), with no statistical difference between the two groups (Table 3). Concomitant anemia and hypoalbuminemia were recorded in three dogs, including one with severe regenerative anemia (Hg: 6.5 g/dL), one with mild regenerative anemia (Hg: 11.1 g/dL), and one with moderate non-regenerative anemia (Hg: 10.1 g/dL). A weak but still statistically significant negative correlation was observed between the FE pattern and the serum albumin concentration (r = −0.52; *p* = 0.01).

Hyponatremia (median 130.5 mmol/L, ranging from 118 to 139; mean of 129.5 mmol/L ± 10.6) was documented in four dogs (28%; three from G1 and one from G2). Concomitant hyperkalemia (6.5 mmol/L and 6 mmol/L) was detected in two dogs (14%; one from each group), with a Na/K < 24 and normal ACTH-stimulation test results, consistent with pseudo-hypoadrenocorticism. Diarrhea was reported in only one of these dogs, while vomiting was not observed. One of these dogs presented with chronic clinical signs. Venous blood gas analysis was available for one of these dogs, revealing metabolic acidosis (pH 7.26, RI 7.35–7.45; HCO 19 mmol/L, RI 19–24; PCO 44 mmHg, RI 40–45).

Plasma chloride concentration was available for three out of four dogs with concomitant hyponatremia, with a median serum chloride concentration of 96 mmol/L, ranging from 90 to 98 (mean of 94.7 mmol/L ± 4.2), and a median corrected serum chloride concentration of 107 mmol/L, ranging from 103 to 119 (mean of 110 ± 8.3).

No correlation was found between the FE pattern and the serum sodium concentration (r = −0.07; *p* = 0.8) or potassium concentration (r = 0.4; *p* = 0.17). However, both dogs with pseudo-hypoadrenocorticism exhibited massive FE.

### 3.6. Abdominal Ultrasound

Abdominal ultrasound was performed in eleven dogs (all from G1), revealing an increased thickness of the intestinal wall (>1.5 mm) in six of them (54%) and an enlargement of the abdominal lymph nodes in four (36%) cases. Intussusception was detected in two dogs from G1, both exhibiting massive FE.

## 4. Discussion

This study identified a small number of dogs diagnosed with *T. vulpis* infection over a 16-year period. Such observation suggests a potentially low prevalence of whipworms in the region where the study was conducted, in accordance with findings from a recent study indicating a relatively low prevalence of *T. vulpis* in dogs in France [10]. Furthermore, the small number of included dogs (n = 45) and the even smaller number of dogs with laboratory abnormalities only permit the suggestion of clinical and laboratory tendencies related to *T. vulpis* infection in dogs, without drafting a strong conclusion.

As previously documented in dogs with trichuriasis, eggs of Ancylostomatidae and eggs of *T. vulpis* are commonly found concomitantly [1,2,3,4,5,6,7,8,9,10,11,12,13,14,15,16,17,18,19,20,21,22,23,24]. This frequent co-occurrence of *T. vulpis* with other intestinal parasites poses a challenge when evaluating the pathogenic influence of whipworms in dogs. In the present investigation, weight loss emerged as the sole clinical sign significantly more prevalent in poly-infected dogs compared to those solely infected with *T. vulpis*. No other signs suggesting heightened pathogenicity were identified in poly-infected dogs.

Numerous individual host factors, such as age, breed, lifestyle, environment, and immune status, have been shown to influence the clinical progression of parasitic illnesses [1,13,25,26,27]. The age of dogs has been proposed as a major factor influencing the presence and severity of clinical signs associated with *T. vulpis* [1,3,15]. In this study, only diarrhea exhibited a higher frequency in young dogs compared to adults, suggesting that GI signs associated with *T. vulpis* infection may manifest in dogs of all ages. Adult dogs were predominant in our study (73%), with the highest FE pattern. A statistically significant positive correlation was found between the age of the dogs and the FE, suggesting a potential increase in FE intensity with age, as previously reported [1,23,28].

Pseudo-hypoadrenocorticism is the most severe clinical manifestation observed in *T. vulpis*-infected dogs, as reported in previous studies, and evidenced in two dogs here described [16,17,18,19,29,30]. The pathogenesis of electrolyte abnormalities associated with GI diseases is complex, and a selective aldosterone deficiency does not appear to account for these abnormalities during *T. vulpis* infection [30]. Dehydration resulting from the loss of isotonic fluids due to diarrhea leads to the replacement of hypotonic fluids with water intake, resulting in sodium content dilution [16]. Additionally, the loss of bicarbonates in stool may induce metabolic acidosis, leading to the shifting of intracellular potassium towards the extracellular space [31,32]. In the present study, despite a low median serum sodium concentration, the median and mean corrected serum chloride concentrations were within the normal range, suggesting that the dilution of sodium content secondary to increased plasma-free water was likely the primary factor contributing to hyponatremia. Concomitant hyperkalemia may be attributed to metabolic acidosis, with the latter being diagnosed in one hyperkalemic dog, based on available venous blood gas analysis results. Nevertheless, as only two dogs presented pseudo-hypoadrenocorticism in the present study, such suggestions remain hypothetical.

In human patients, *Trichurs trichiura* is known to cause colitis and iron-deficiency anemia, and both moderate and high-intensity *T. trichiura* infections have been associated with anemia [33,34,35]. The hematophagous behavior of *T. vulpis* was first suggested in 1964 with the discovery of red blood cells in the esophagus of the parasite and positive benzidine reaction tests of adult whipworms [36]. In naturally infected dogs, a decrease in Hg concentration and a negative correlation between the FE pattern and Hg value have also been reported [37]. In a previous case series, moderate anemia was observed in around 30% (4/13) of dogs naturally infected with *T. vulpis*. However, in that study, two dogs had concomitant diseases (portosystemic shunt and pancreatitis), complicating the evaluation of a potential relationship between anemia and *T. vulpis* infection [17]. Since *A. caninum* is a well-known voracious hematophagous intestinal parasite, its primary role in anemia has been suggested in *T. vulpis*/*A. caninum* co-infected dogs [14,20,38]. In the study discussed herein, anemia was documented in 23% of dogs with available CBC results, with a similar frequency observed between mono- and poly-infected dogs. The median and mean Hg concentrations were also comparable between mono- and poly-infected dogs, and severe anemia was only noted in mono-infected dogs. These findings suggest that the presence and severity of anemia associated with *T. vulpis* infection are not solely attributable to co-infections with hematophagous parasites like hookworms. While the possibility of a missed diagnosis of concomitant hookworm infection in dogs from G1 cannot be entirely ruled out, it appears unlikely given that the fecal flotation sensitivity for detecting Ancylostomatidae eggs is equal to, if not better than, that for *T. vulpis* [39]. Overall, regenerative anemia was observed in 57% of dogs, suggesting that GI blood loss may contribute to its onset. Microcytosis was noted in two anemic dogs, potentially indicating chronic blood loss. Assessing serum iron levels and transferrin saturation percentage could have provided valuable insights into anemia secondary to GI blood loss. Unfortunately, these parameters were not measured in either our cohort or previous studies. Hence, while *T. vulpis* may contribute to the development of anemia, the precise mechanism remains unclear. Furthermore, the low number of dogs presenting anemia and the absence of available CBC results for some dogs do not permit a strong conclusion. Additionally, although a correlation between the FE pattern and Hg concentration has been previously described [37], such an association was not observed in the current study.

Consistent with previous investigations on dogs infected with *T. vulpis* [13], leukocytosis was also a common finding among dogs in the present study (30%). However, CBC results were not available in all dogs, potentially biasing these results. *Trichuris muris* infection in mice has served as a widely utilized experimental model for exploring the interactions between hosts and nematodes of the genus *Trichuris*. A previous study has documented an elevation in leukocyte counts in mice subjected to experimental *T. muris* infection, indicating an immunological reaction to *T. muris*-induced tissue damage or potential intestinal bacterial translocation [40].

This study also documented abdominal US abnormalities in dogs with *T. vulpis* infection. The observed US findings, such as the thickening of the intestinal wall (54%) and the enlargement of abdominal lymph nodes (36%), lack specificity, as they are commonly observed in dogs with various chronic digestive disorders [41]. Interestingly, intussusception was identified in two dogs from G1, both of which presented with massive FE patterns. While causality cannot be confirmed, these findings suggest a possible relationship between intussusception and a significant parasitic burden. Nevertheless, only 11 dogs (all from G1) had abdominal US performed. Therefore, it is not possible to draft a definitive conclusion, and such findings remain to be confirmed.

This study has several limitations. Fecal egg quantification is usually conducted using the McMaster or Kato–Katz thick smear techniques [42,43,44]. However, in the present study, only a qualitative methodology was employed, leading to an approximate estimation of the egg count in the feces. Additionally, the shedding of *T. vulpis* eggs is intermittent, and the FE pattern may not accurately reflect the adult burden in the host’s large intestine [3]. Furthermore, a standardized stool sample collection was not implemented. The presence of different intestinal parasitic species may influence the host–parasite species composition, altering both richness and alpha diversity [45,46]. Nevertheless, no difference was noted when comparing the FE pattern between mono-infected and poly-infected dogs in this study. Therefore, a negative impact on the FE pattern by multiple parasitic infections seems unlikely in the examined cohort. Also, the differentiation between different species of hookworms was not conducted in the present study, as only a morphological analysis of the Ancylostomatidae eggs found in the feces was performed. Finally, as in any retrospective study, there is a possibility that the frequency of clinical and laboratory abnormalities may have been incorrectly estimated, and clinical and biological data were incomplete for some dogs in this study. Moreover, the considerable period of 16 years may have affected the clinical and lifestyle factors of the dogs. Additionally, during this extended period, both the instruments and methodologies have evolved, along with the medical focus on certain clinical signs and laboratory abnormalities (including US findings). Therefore, results from this study must be interpreted accordingly.

## 5. Conclusions

The presence of GI signs and laboratory abnormalities in both mono-infected and poly-infected dogs and their overall similar frequency suggest that *T. vulpis* might contribute to the observed clinical and diagnostic findings. Furthermore, six dogs exhibited severe clinical conditions such as pseudo-hypoadrenocorticism, intussusception, and severe anemia, further suggesting a pathogenic role of this nematode. Hypoalbuminemia and abnormalities at CBC were frequently observed, with a statistically significant correlation between the FE pattern and leukocytosis, lymphopenia, and hypoalbuminemia, also suggesting a potential relation between the parasitic burden and the severity of laboratory abnormalities. Anemia was detected in five dogs infected with *T. vulpis* alone, and in two of them, it was severe. Although the FE pattern showed a positive correlation with the age of the dogs, both young and adult dogs presented with a similar clinical picture overall, indicating that dogs of all ages can exhibit clinical signs associated with *T. vulpis* infection. However, given the large inclusion period (16 years), the small number of dogs, the lack of laboratory and US results in some animals, and the subjective and semi-quantitative method for the estimation of the FE pattern, results from this study only permit the suggestion of clinical and laboratory tendencies related to *T. vulpis* infection in dogs, without draft strong conclusion. Prospective studies are needed to gain a better understanding of the clinical behavior of *T. vulpis*, identify risk factors related to its clinical expression, and elucidate the mechanism underlying severe clinical syndromes potentially associated with whipworm infection in dogs.

## Figures and Tables

**Table 1:** Endoparasites other than *T. vulpis* found in poly-infected dogs from Group 2, and the prevalence of co-infections both across all 45 included dogs and among the 20 poly-infected dogs (Group 2).

Concomitant Parasitic Infections	Number of Infected Dogs	Group 2 (n = 20)	All Dogs (n = 45)
*Toxocara canis*	11	55%	24%
Ancylostomatidae	8	40%	18%
*Cystoisospora* spp.	7	35%	15%
*Giardia duodenalis*	4	20%	9%
*Eucoleus aerophilus*	2	10%	4%
Taeniidae	1	5%	2%

**Table 2:** The frequency of clinical signs in dogs solely infected with *T. vulpis* (Group G1) and in poly-infected dogs (Group G2) at the time of initial presentation.

Clinical Signs	Group 1 (G1)	Group 2 (G2)	Overall	*p* Value (GI versus G2)
Diarrhea	40% (10/25)	60% (12/20)	49% (22/45)	0.18
Weight loss	20% (5/25)	60% (12/20)	38% (17/45)	0.006 *
Hematochezia	28% (7/25)	25% (6/20)	29% (13/45)	0.88
Hyporexia	28% (7/25)	30% (6/20)	29% (13/45)	0.88
Vomiting	16% (4/25)	20% (4/20)	18% (8/45)	0.73
Polyphagia	12% (3/25)	5% (1/20)	9% (4/45)	0.41
Others	24% (6/25)	10% (2/20)	18% (8/45)	0.27
None	4% (1/25)	10% (2/20)	7% (3/45)	0.58

Values of *p* < 0.05 (*) were considered significant.

**Table 3:** Laboratory findings, including complete blood count and biochemistry results from all dogs upon initial presentation. No difference was found between *T. vulpis* mono-infected and poly-infected dogs.

Parameters	Reference Interval	Overall Range	Overall Median	Overall Mean ± SD	Group 1 Mean ± SD	Group 2 Mean ± SD	*p* Value (G1 versus G2)
Complete blood count ^1^							
Hemoglobin (g/dL)	12–18	6.5 to 17.8	13.9	13.3 ± 3.4	13.6 ± 3.3	12.7 ±3.7	0.27
Hematocrit (%)	37–54	19.6 to 53.6	40.7	39.6 ± 9.8	39.2 ± 10.5	40.6 ± 8.7	0.36
RBC (M/mm^3^)	5.5–8.5	2.8 to 7.8	5.9	5.8 ± 1.28	5.7 ± 1.4	6.0 ± 1	0.26
MCV (fL)	60–71	45 to 78	69	68.2 ± 6.8	68.7 ± 6.9	67.2 ± 6.8	0.36
Reticulocytes (×10^9^/L)	<60	0 to 1015	105	108.8 ± 33.6	94.8 ± 43	29 ± 41	0.14
WBC (m/mm^3^)	6–17	7.1 to 57.0	15.1	17.8 ± 12.7	17.5 ± 11.8	18.4 ± 15	0.43
Neutrophils (m/mm^3^)	2.9–13.6	5.4 to 49.1	11.7	14.1 ± 9.7	15.4 ± 11	11.2 ± 5.1	0.15
Monocytes (m/mm^3^)	0.3–1.6	0 to 5.6	0.4	0.68 ± 1.1	0.8 ± 1.3	0.4 ± 0.4	0.78
Lymphocytes (m/mm^3^)	1.1–5.3	0.3 to 3.3	1.1	1.3 ± 0.8	1.2 ± 0.8	1.4 ± 0.9	0.30
Eosinophils (m/mm^3^)	0–0.5	0 to 1.9	0.2	0.4 ± 0.6	0.4 ± 0.6	0.4 ± 0.6	0.42
Platelets (m/mm^3^)	140–600	171 to 978	335	382 ± 194	391 ± 224	364 ± 127	0.37
Biochemistry ^2^							
Albumin (g/L)	27–36	18 to 38	26	26.3 ± 4.5	26.6 ± 3.5	25.6 ± 6.4	0.31
Sodium (mmol/L)	142–152	118 to 170	146.5	145.6 ± 13.6	146 ± 13	144 ± 18.5	0.41
Potassium (mmol/L)	3.6–5.6	3.1 to 6.5	3.9	4.3 ± 1	4.3 ± 1	4.4 ± 1.4	0.45
Measured chloride (mmol/L) ^3^	104–112	90 to 133	114	110 ± 11.2	112 ± 10.6	105 ± 13.7	0.18

^1^ RBC indices available in 27 dogs and WBC differential count in 26 dogs. ^2^ Biochemistry results available in 21 dogs (including serum electrolytes concentration in 14 dogs). ^3^ Results available in 13 dogs. Values of *p* < 0.05 were considered significant.

## Data Availability

The original contributions presented in the study are included in the article, and further inquiries can be directed to the corresponding author.
